# Tuberculosis exposure, infection and disease in children: a systematic diagnostic approach

**DOI:** 10.1186/s41479-016-0023-9

**Published:** 2016-11-24

**Authors:** Claudia L. Roya-Pabon, Carlos M. Perez-Velez

**Affiliations:** 1 Division of Pediatric Pulmonology, Department of Pediatrics, Faculty of Medicine, University of Antioquia, Medellin, Antioquia Colombia; 2 Grupo Tuberculosis Valle-Colorado (GTVC), Medellin, Antioquia Colombia; 3 Tuberculosis Clinic, Pima County Health Department, Tucson, AZ USA; 4 Division of Infectious Diseases, College of Medicine, University of Arizona, Tucson, AZ USA; 5 College of Medicine, University of Arizona, 1501 North Campbell Avenue, P.O. Box 245039, 85724 Tucson, AZ USA

**Keywords:** Latent tuberculosis, Algorithm, Diagnostic techniques and procedures, Specimen handling, Risk factors

## Abstract

The accurate diagnosis of tuberculosis (TB) in children remains challenging. A myriad of common childhood diseases can present with similar symptoms and signs, and differentiating between exposure and infection, as well as infection and disease can be problematic. The paucibacillary nature of childhood TB complicates bacteriological confirmation and specimen collection is difficult. In most instances intrathoracic TB remains a clinical diagnosis. TB infection and disease represent a dynamic continuum from TB exposure with/without infection, to subclinical/incipient disease, to non-severe and severe disease. The clinical spectrum of intrathoracic TB in children is broad, and the classification of clinical, radiological, endoscopic, and laboratory findings into recognized clinical syndromes allows a more refined diagnostic approach in order to minimize both under- and over-diagnosis. Bacteriological confirmation can be improved significantly by collecting multiple, high-quality specimens from the most appropriate source. Mycobacterial testing should include traditional smear microscopy and culture, as well as nucleic acid amplification testing. A systematic approach to the child with recent exposure to TB, or with clinical and radiological findings compatible with this diagnosis, should allow pragmatic classification as TB exposure, infection, or disease to facilitate timely and appropriate management. It is important to also assess risk factors for TB disease progression and to undertake follow-up evaluations to monitor treatment response and ongoing evidence supporting a TB, or alternative, diagnosis.

**Electronic supplementary material:**

The online version of this article (doi:10.1186/s41479-016-0023-9) contains supplementary material, which is available to authorized users.

## Background

Diagnosing tuberculosis (TB) in children is challenging (Table 1) [1] and often it is only considered after the child has failed various therapeutic trials for other disorders. Even with intensive specimen collection and optimal molecular and culture-based diagnostics, most children with non-severe pulmonary TB are not confirmed bacteriologically, despite having an exposure history, immune-based confirmation of infection and clinical features consistent with this diagnosis [2]. Nonetheless, with currently available tools, it is possible to make an accurate clinical diagnosis of intrathoracic TB in most diseased children. This review presents a systematic approach to diagnosing intrathoracic TB in children.

**Table 1:** Challenges in diagnosing TB exposure, infection and disease in children

Disease state	Main challenges	Current status & limitations	Recent advances & future prospects
Infection	Differentiating between TB exposure (without infection), and TB infection	Current immune-based tests (TST and IGRAs) may not convert to positive until 2–10 weeks after acquiring *M. tb* infection	Mycobacteria-specific cytokine biomarkers -- alone or in combination (i.e., biosignatures) -- may distinguish between TB exposure (without infection), and TB infection [90]
	Differentiating between infection and subclinical disease	Chest radiography is the first-line imaging modality, but may not reveal abnormalities consistent with TB disease in all cases -- especially those in early states of the continuum of TB	Chest CT, MRI, and PET [91] scan may reveal findings consistent with TB disease before symptoms develop
Disease	Detection of TB disease and of drug resistance	Currently available immune-based tests (TST and IGRAs) do not differentiate between infection and disease	Mycobacteria-specific cytokine biomarkers -- alone or in combination -- may distinguish between TB infection and TB disease [90]
		Currently available tests (e.g. NAATs; culture) for bacteriological confirmation have limited sensitivity for detecting *M. tb* in young children with paucibacillary disease--especially in early states of the continuum of TB	- Xpert MTB/RIF Ultra (Cepheid): next generation, ultrasensitive NAAT for detection of both *M. tb* & rifamycin resistance; *in vitro* study demonstrated sensitivity comparable to culture [92, 93]. - GeneXpert Omni (Cepheid): single-cartridge battery-operated platform that is portable/mobile; study pending [93] - Xpert XDR NAAT (Cepheid): study anticipated in 2018 [93]
		Specimen collection for bacteriological confirmation currently consists of serial sampling of three gastric aspirates/lavages or induced sputa and requires trained personnel and facilities with airborne infection control	Strategies consisting of “intensive” collection of combinations of various specimens (e.g., nasopharyngeal aspirates; string tests; stool; fine needle aspirate of diseased lymph node) that have similar or superior bacteriological yield, require less training, and may be carried out as an outpatient over 1–2 days
	Monitoring response to treatment	Mycobacterial culture is only useful in those children who had positive cultures at time of diagnosis (minority of cases).	Cytokine biomarkers and biosignatures (possibly including IFN-γ, TNF-α, IL-2, IL-6, IL-10 and/or IL-12) [94, 95]
			18 F-FDG PET/CT is sensitive for the detection of TB disease (in different states of the continuum) and for monitoring response to treatment [91]

*CT* computed tomography, *IGRA* interferon-gamma release assay, *MRI* magnetic resonance imaging, *M. tb:* Mycobacterium tuberculosis, *NAAT* nucleic acid amplification test, *PCR* polymerase chain reaction, *PET* positron emission tomography, *TB* tuberculosis, *TST* tuberculin skin test, *XDR* extensively drug-resistant

### Continuum of TB states

**Fig. 1:**
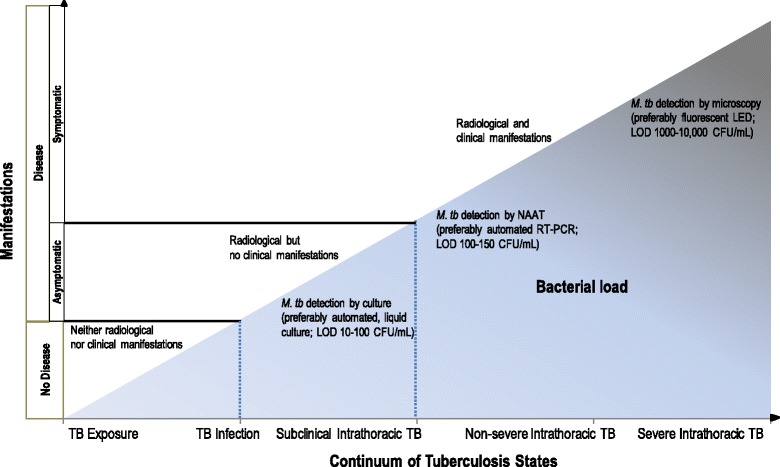
Continuum of TB states and correlations with bacterial load and with radiological and clinical manifestations. CFU: colony-forming units; LED: light-emitting diode; LOD: limit of detection; mL: milliliter; NAAT: nucleic acid amplification test; RT-PCR: real-time polymerase chain reaction. Adapted from C.M. Perez-Velez. Diagnosis of Intrathoracic Tuberculosis in Children. In: Handbook of Child and Adolescent Tuberculosis (p. 149), J.R. Starke and P.R. Donald (Eds.), 2016, New York, NY: Oxford University Press. Copyright by Oxford University Press [15]. Adapted with permission

Although much remains unknown about its pathophysiology, TB studies characterized a dynamic continuum of various states that include exposure, infection, subclinical or incipient disease, non-severe and severe disease states (Fig. 1) [3, 4]. Generally, this continuum correlates with bacterial burden [5]. As the archetypical human pathogen, *Mycobacterium tuberculosis* establishes a sustained but “delicately balanced” host–pathogen relationship [6]. These TB states depend upon various host (e.g. immunological competence), pathogen (e.g. strain virulence), and environmental (e.g. intensity of exposure) factors. The clinical outcome of infection will thus be either self-cure, latency or disease [7]. Understanding that TB is a continuum of states—and not a dichotomy of infection or disease—has important implications for managing children in whom latent or active TB often cannot be confirmed.

### Clinical spectrum of disease

Once infected with *M. tuberculosis*, young children (aged <5 years) are at greater risk than adults of progressing to disease, including its most severe forms. This depends on the child’s susceptibility, which is highest during the first years of life, probably from immunological immaturity. Without Bacille Calmette-Guerin (BCG) vaccination, approximately 30% of infected infants (<1 year old) will progress to intrathoracic TB, and 10–20% will develop disseminated disease. In children aged 1–2 years, the risk of progressing to intrathoracic TB is 10–20 and 2–5% for disseminated disease. These risks decline slowly until around 10 years of age when adult-type disease starts to emerge [8, 9]. Thus, early diagnosis is important, especially in infants and young children who are at greatest risk of rapid disease development [8] and clinicians should consider the full clinical spectrum of intrathoracic syndromes [10].

### Clinical classification of tuberculosis

Classifying intrathoracic TB by immunopathogenesis (Table 2) assists understanding how each possible “state” in the continuum is managed [11]. For example, a child with a history of TB exposure can have the features of subclinical disease [12] outlined in Table 2, which in some hierarchical diagnostic classification systems corresponds to “possible” intrathoracic TB. A typical example is that of a young child with isolated uncomplicated hilar lymphadenopathy [13]. Such a child may not meet sufficient criteria to be clinically diagnosed with “probable” intrathoracic TB given their lack of symptoms and physical signs, [14] and consequently may not receive treatment for tuberculosis disease or infection. Whether this intermediate state will progress to clinically manifest disease or be contained as latent infection is dependent on the child’s level of immunocompetence. In those with risk factors for progression to TB disease, treatment is recommended. Children with disease can be further classified as severe or non-severe, depending on whether or not infection is contained and on the presence and extent of complications.

**Table 2:** Clinical classification of intrathoracic TB based on immunopathogenesis

Clinical classification	Immunopathogenesis	TST/IGRA	Imaging	Clinical manifestations	Myco-bacterial detection
TB exposure	Self-cure (*infection eliminated* by innate immune response; no T-cell activation)	Negative	Normal	None	Negative
Latent TB infection	Quiescent infection (*non-replicating bacteria* persisting with very low metabolic activity; infection well-contained)	Positive	- Calcified non-enlarged regional lymph nodes - Calcified lung nodules - Pleural thickening	None	Negative
Subclinical TB	Incipient disease (*replicating bacteria* that are metabolically active; *infection contained*)	Usually positive	- Uncomplicated hilar/mediastinal lymphadenopathy - Non-calcified lung nodules - Uncomplicated pleural effusion	None	Usually negative (may be transiently positive)
Non-severe TB	Mild-to-moderate disease (replicating bacteria that are metabolically active; *infection only partially contained*)	Usually positive	- Uncomplicated hilar/mediastinal lymphadenopathy - Non-calcified lung nodules - Uncomplicated pleural effusion	Mild-to-moderate	Positive cultures (10–30% of cases)
Severe TB	Severe disease (replicating bacteria that are metabolically active; *infection not contained*)	Usually positive	See spectrum of disease (Fig. 2)	Severe	Positive cultures (30–70% of cases)

Adapted from C.M. Perez-Velez. Diagnosis of Intrathoracic Tuberculosis in Children. In: Handbook of Child and Adolescent Tuberculosis (p. 149), J.R. Starke and P.R. Donald (Eds.), 2016, New York, NY: Oxford University Press. Copyright by Oxford University Press [15]. Adapted with permission

*IGRA* Interferon-gamma release assay, *PCR* polymerase chain reaction, *TB* tuberculosis, *TST* tuberculin skin test

### Systematic diagnostic approach

As it is impossible to achieve bacteriological confirmation in many childhood TB cases, systematically identifying findings suggestive of TB can allow for its clinical diagnosis. Excluding other differential diagnoses and observing a positive therapeutic response increases the probability of TB being the correct diagnosis. The following systematic approach to diagnosing TB in children consists of (i) identifying findings suggesting TB disease; (ii) identifying findings supportive of TB as the etiology; (iii) screening for risk factors for progression to disease; and (iv) follow-up evaluations to further support or exclude TB as the etiology (Table 3) [15].

**Table 3:** Systematic approach to the diagnosis of intrathoracic TB in children

Step 1: Identify findings suggestive of TB disease
• Clinical evaluation: history & physical exam
• Radiological imaging: chest radiography; computed tomography; ultrasonography
• Laboratory studies: composite measures (cell count and chemistry) of body fluids (e.g., pleural fluid)
• Endoscopic studies: bronchoscopy
Step 2: Identifying findings supportive of TB as the etiology
• TB exposure history
• Immune-based tests: TST; IGRA
• Biochemical markers: ADA in body fluids (e.g., pleural fluid; pericardial fluid)
• Mycobacterial detection: smear microscopy; NAAT; culture; antigen test (in HIV-infected adolescents, lateral flow lipoarabinomannan in urine with CD4 < 100)
• Histopathological & cytopathological studies
• Excluding other differential diagnoses
Step 3: Screen for risk factors for progression to TB disease
• Age groups (e.g. immunological immaturity of infancy)
• Immunocompromising conditions (e.g., HIV infection)
• Immunosuppressive medications (e.g., TNF-α) antagonists
• Contained TB infection-disease (e.g., noncalcified fibronodular lesions, especially apical, on chest imaging
• Environment (e.g., continued exposure)
Step 4: Follow-up evaluation to support or exclude TB as the etiology

Adapted from C.M. Perez-Velez. Diagnosis of Intrathoracic Tuberculosis in Children. In: Handbook of Child and Adolescent Tuberculosis (p. 149), J.R. Starke and P.R. Donald (Eds.), 2016, New York, NY: Oxford University Press. Copyright by Oxford University Press [15]. Adapted with permission

*ADA* adenosine deaminase, *CD4* cluster of differentiation 4, *HIV* human immunodeficiency virus, *IGRA* interferon gamma release assay, *NAAT* nucleic acid amplification test, *TB* tuberculosis, *TNF-α* tumor necrosis factor alpha, *TST* tuberculin skin test

## STEP 1: Identify findings suggestive of TB disease

Each intrathoracic clinical syndrome of TB disease has its own constellation of clinical, radiological, laboratory, and endoscopic (if indicated) findings, although many are shared by more than one clinical syndrome. Furthermore, miliary lung disease may also involve potentially any organ system (Additional file 1: Textbox 1). Most clinical manifestations of intrathoracic TB result from the overall balance of beneficial and harmful immune responses to *M. tuberculosis* and a severe inflammatory reaction can be triggered by a relatively low burden of organisms. There are no clinical features pathognomonic of intrathoracic TB, but combinations of symptoms and physical signs with certain temporal patterns can help differentiate it from other etiologies that might mimic this disorder.

### Clinical evaluation

Pulmonary TB is frequently associated with intrathoracic lymphadenopathy, and sometimes with pleural or pericardial disease, and therefore “intrathoracic TB” is the preferred term in children. Localized symptoms and physical signs depend on which intrathoracic organs are involved, while non-localized symptoms and signs are independent of the organ-specific clinical syndrome. Symptoms and physical signs that are well-defined have higher specificity. However, in children who are immunocompromised (e.g. less than three years of age with immunological immaturity), HIV-infected, or severely malnourished, these symptoms and signs have lower sensitivity and specificity [16].

Systemic symptoms and signs may appear early or late in the disease course [17]. Daily fever is characteristically >38.0 ° C, intermittent or persistent throughout the day, and usually lasts >1 week. Night sweats are uncommon, subjective and nonspecific, and are significant only when they drench the child’s clothes and bedding. Chills and rigors are rare, except in disseminated disease. Anorexia and associated wasting or failure to thrive during the past 3–6 months, or having lost >10% of body weight over any interval of time, are sensitive—albeit nonspecific—signs in most TB clinical syndromes in young children [16]. The immunocompromised state from severe under-nutrition can increase the risk for a paradoxical reaction when they receive TB treatment and nutritional rehabilitation [18]. Fatigue, asthenia, and malaise may manifest in young children as listlessness (e.g. decreased playfulness) and in infants as apathy (e.g. less interactive with caregivers) and should be persistent and not attributable to other causes.

Peripheral lymphadenopathy from TB typically consists of a unilateral, enlarged, non-painful, rubbery lymph node, sometimes becoming fluctuant, with or without spontaneous drainage forming a sinus tract [19]. Respiratory symptoms and signs depend on the site, and degree of involvement (e.g. of airway obstruction). The cough is usually unremitting for >2 weeks and may be “dry” or “wet”. When the airway is compressed by an enlarged lymph node, there may be persistent cough, wheezing or stridor that does not improve with inhaled bronchodilators (Additional file 1: Table S1). Characterizing the temporal pattern (including the onset, progression and duration) of symptoms helps clinicians to identify cases with likely intrathoracic TB.

### Radiological Imaging

Chest imaging—including radiography, computed tomography (CT), and ultrasonography—is one of the most useful diagnostic modalities for detecting intrathoracic TB. The spectrum of radiological abnormalities in children is very broad, and none are sufficiently specific to confirm the diagnosis [20, 21]. Nonetheless, certain patterns and signs are highly suggestive, especially when accompanied by clinical features and supportive findings (e.g. recent TB exposure, and positive T-cell-based test). Recognizing such radiological patterns (Fig. 2) helps narrow the differential diagnosis (Additional file 1: Table S3). Chest radiography—including both frontal and lateral projections—is the first-line imaging modality when intrathoracic TB is suspected. The lateral projection helps detect retrocarinal, subcarinal, and superimposed hilar lymphadenopathy, especially in infants where the thymus may obscure enlarged nodes on the frontal view [22, 23]. Additionally, CT scans may detect abnormalities suggestive of intrathoracic TB in a child suspected of having complicated intrathoracic lymph node or pleural disease, endobronchial lesions, bronchiectasis, or cavities that are not well revealed on plain radiography [24, 25]. Finally, chest ultrasonography is useful for evaluating mediastinal lymphadenopathy and pericardial effusions. Also, it is the preferred imaging modality in differentiating loculated from free-flowing pleural effusions [26, 27].

**Fig. 2:**
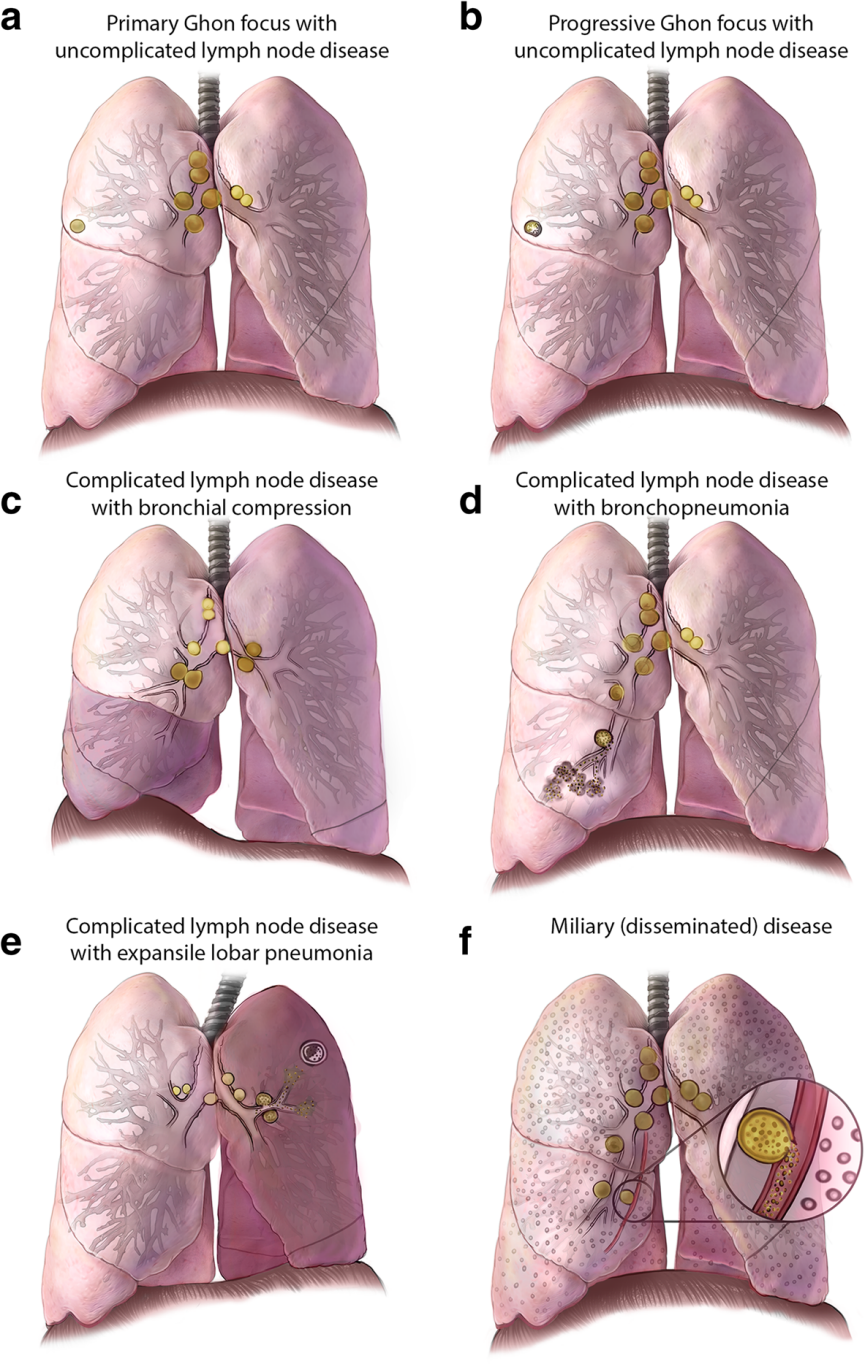
Illustrations of radiological patterns caused by intrathoracic TB in children. Panel **a**. Primary Ghon focus with uncomplicated lymph node disease. Hilar and mediastinal lymphadenopathy associated with an ipsilateral peripheral nodule, or “Ghon focus” (*right lung*); these nodules are often subpleural with an overlying pleural reaction. Panel **b**. Progressive Ghon focus with uncomplicated lymph node disease. A Ghon focus with cavitation (*right lung*), which is seen almost exclusively in infants and immunocompromised children; other elements of the Ghon complex are also visible. Panel **c**. Complicated lymph node disease with bronchial compression. Enlarged lymph nodes compressing the airway, causing either complete obstruction with lobar collapse (*right middle and lower lobes*), or partial obstruction with a ball-valve effect leading to hyperinflation *(left upper and lower lobes*). Panel **d**. Complicated lymph node disease with bronchopneumonia. Necrotic lymph nodes erupting into bronchus intermedius, with endobronchial spread and patchy consolidation of the middle lobe (*right lung*). Panel **e**. Complicated lymph node disease with expansile lobar pneumonia. Necrotic lymph nodes that compress and obstruct the left upper lobe bronchus and may infiltrate a phrenic nerve, causing hemidiaphragmatic palsy (*left-sided*); endobronchial spread causes dense consolidation of the entire lobe (*left upper lobe*), with displacement of the trachea and fissures and the formation of focal cavities. Panel **f**. Miliary (disseminated) disease. Diffuse micronodules in both lungs, which may result from lymphohematogenous spread after recent primary infection or from infiltrating a necrotic lymph node or lung lesion into a blood vessel, leading to hematogenous spread

**Fig. 2:**
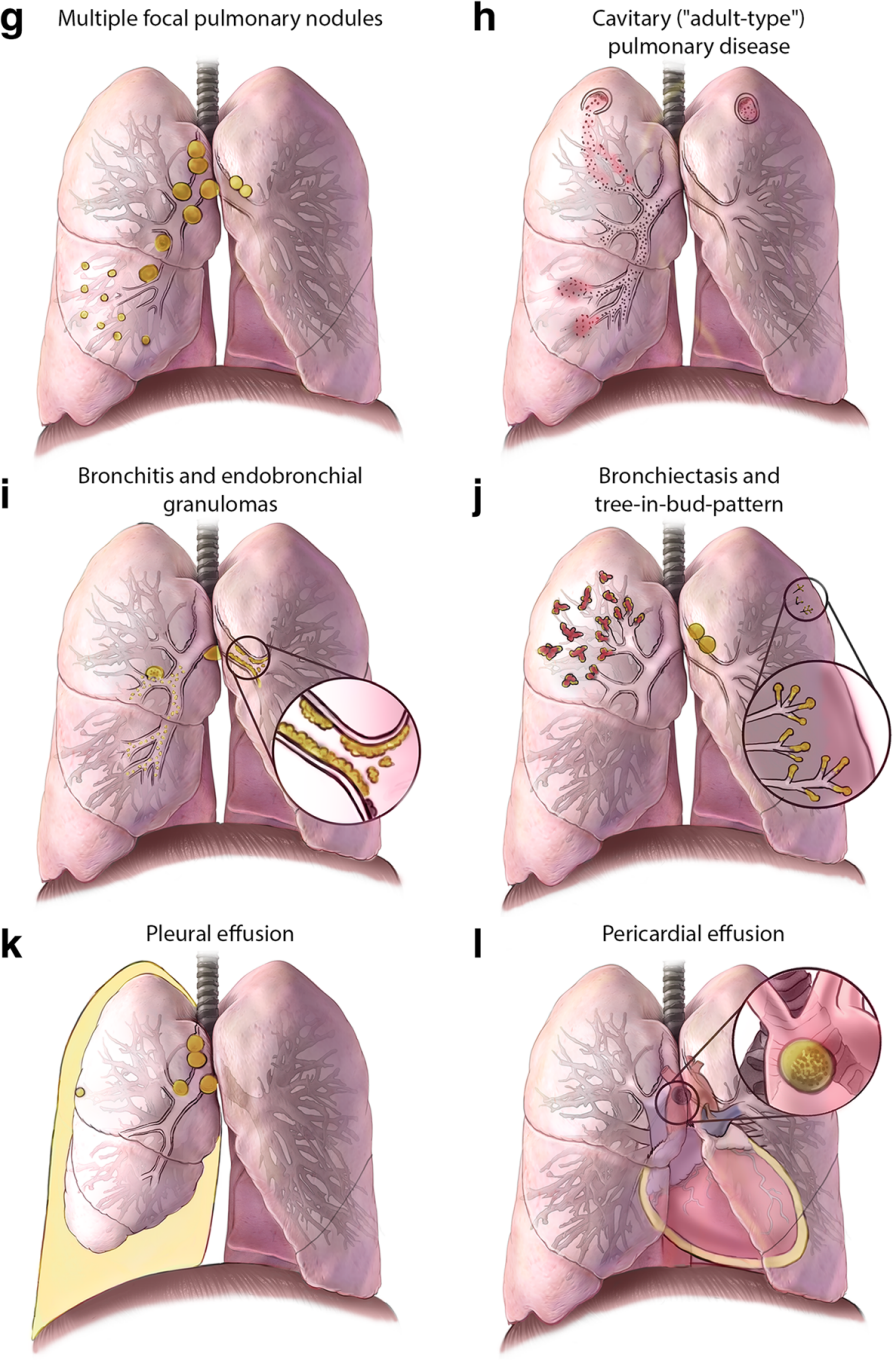
Panel **g**. Multiple focal pulmonary nodules. Multiple focal pulmonary nodules involving the right middle lobe with enlargement of regional lymph nodes (*right lung*). Panel **h**. Cavitary (“adult-type”) pulmonary disease. Cavity formation in both upper lobes, with endobronchial spread to the right middle lobe. Nodules or cavities in apical lung segments are typical of adult-type disease and are pathologically distinct from the other cavities shown. Panel **i**. Bronchitis and endobronchial granulomas. Inflammation of the mucosa of main stem bronchus with purulent secretions (*left lung*), and a necrotic lymph node that has eroded into the right middle lobe bronchus leading to endobronchial spread and subsequent development of endobronchial granulomas extending proximally to the bronchus intermedius and main stem bronchus, and distally to the lower lobe bronchus (*right lung*). These findings are best visualized by bronchoscopy. Panel **j**. Bronchiectasis and tree-in-bud-pattern. Bronchiectasis that extensively involves the upper lobe (*right lung*), and shows tree-in-bud pattern observable on CT scans -- reflecting dilated centrilobular bronchioles with mucoid impaction -- involving the upper lobe (*left lung*). Panel **k**. Pleural effusion. A pleural effusion that is usually indicative of recent primary infection, with a hypersensitivity response to tuberculoprotein leaking from a subpleural Ghon focus (often not visible) into the pleural cavity; in rare cases this effusion may also result from a chylothorax. Panel **l**. Pericardial effusion. A pericardial effusion that occurs when tuberculoprotein leaks from a necrotic subcarinal lymph node (shown in “close-up” window) into the pericardial space; it may also occur after hematogenous spread. Conceptualization and original sketches by C.L. Roya-Pabon, MD; finished artwork by Mesa Schumacher, MA (used with permission). Adapted from C.M. Perez-Velez. Diagnosis of Intrathoracic Tuberculosis in Children. In: Handbook of Child and Adolescent Tuberculosis (p. 154–155), J.R. Starke and P.R. Donald (Eds.), 2016, New York, NY: Oxford University Press. Copyright by Oxford University Press [15]. Adapted with permission

### Endoscopy

Bronchoscopy may be useful in diagnosing and managing cases with tracheobronchial disease [28]. However, it is not routinely indicated for evaluating all intrathoracic TB clinical syndromes.

### Laboratory tests

While non-microbiological laboratory tests results can suggest TB disease, they are not confirmatory of this diagnosis. The cell count and chemistry of body fluids, such as pleural or pericardial fluids, can raise the possibility of TB when the composite results are consistent with the disease. However, other diseases, including non-tuberculous mycobacterial species and fungal infections, can also provide similar results. The following features suggest TB: cell count predominantly lymphocytic (may be neutrophilic within the first few days); elevated protein level (>30 g/L; protein/serum protein ratio >0.5); elevated LDH (>200 IU/L; LDH/serum LDH ratio >0.6); glucose 3.0–5.5 mmol/L (usually lower in effusions due to pyogenic bacteria or rheumatoid arthritis); normal lipids (elevated in chylous effusions). The most common findings on full blood count are mild anemia, neutrophilia, and monocytosis, but these abnormalities are found just as frequently in other respiratory infections [29]. Erythrocyte sedimentation rate may be normal or elevated (e.g. >100 mm/h), but is nonspecific, as are C-reactive protein and procalcitonin [30, 31].

## STEP 2: Identify findings supportive of TB as the etiology

The positive predictive value of each of the following types of findings depends on the local TB prevalence.

### Exposure history

Children are usually infected following exposure to someone with pulmonary TB whose sputum is positive by microscopy or culture, who is actively coughing, and with whom they share the same space (e.g. household, daycare centers, schools, healthcare facilities, refugee camps). In children aged <5 years, the source case is most often from the same household, and the infection usually acquired within the past year. As children become exposed to the community outside the household, their risk of acquiring infection from this source increases and inquiring about confirmed or suspected TB contacts and knowledge of the local TB epidemiology becomes more pertinent [32].

### Immune-based testing

Memory T-cells, detected by a tuberculin skin test (TST) and current *M. tuberculosis* interferon-gamma release assays (IGRAs) measure lasting TB immune responses and can represent any of the following: active TB disease, previous TB disease, latent TB infection, recent or remote TB exposure, or exposure to environmental nontuberculous mycobacteria (NTM; e.g. *M. kansasii*, *M. szulgai*, *M. marinum*) that may have cross-reactivity. Neither IGRAs nor TST can distinguish latent from active TB [33]. Table 4 provides a comparison of currently approved T-cell-based tests including TST, and IGRAs specific for *M. tuberculosis*, such as the ELISPOT-based T-SPOT TB (Oxford Immunotec) and the ELISA-based QuantiFERON (QFT) Gold In-Tube and QFT Gold Plus (Qiagen). TST and IGRAs are complementary, so using both increases sensitivity [42].

**Table 4:** Comparison of T cell-based tests for TB infection

Characteristic	Tuberculin Skin Test	QuantiFERON-TB®*	T-SPOT-TB®**
Time to results	48–72 h	24–36 h	36–48 h
Complexity	Low	Moderate	High
TB antigens	PPD-tuberculin (not specific to *M. tb*.)	ESAT-6; CFP-10; TB-7.7	ESAT-6; CFP-10
Measurement	Skin induration after *in vivo* stimulation	ELISA-based measurement of IFN-γ production by T-cells after *in vitro* stimulation	ELISPOT-based measurement of IFN-γ-producing T-cells (spots) after *in vitro* stimulation
Minimum number of visits to complete testing	2 visits (within 48–72 h of placement)	1 visit	
Sample/Method	Intradermal injection of 5 units of PPD-tuberculin	Blood draw	
Reliability/Variability of test results	Limited variability with appropriate training [34]	Significant within-person variability [35, 36]	
Cross-reactivity with BCG vaccine	Yes (particularly if vaccinated after infancy or repeatedly) [37, 38]	No	
NTM cross reaction	Many	Few (*M. kansasii*, *M. marinum*, *M. szulgaii*)	
Booster effect with repeated testing	Yes	No	
Booster effect after prior TST	Yes	Possible (but likely inconsequential if blood drawn < 3 days after TST [39]	
Internal controls	No	Yes	
Utility by age	Less reliable in children under 6-months of age	Less reliable in children under 5-years of age	
Sensitivity with bacteriologically-confirmed TB	75–85% [40]	80–85% [40]	
Specificity with bacteriologically-confirmed TB	95–100% [40] With BCG vaccination 49–65% [40]	90–95% [40] With BCG vaccination 89–100% [40]	
Sensitivity in HIV-infected patients	45% [36]	Same as TST [36]; T-SPOT.TB slightly less affected by immunosuppression than QFT [41]	

*BCG* bacille Calmette-Guérin, *IGRA* interferon-gamma release assay, *M. tb Mycobacterium tuberculosis*, *NTM* nontuberculous mycobacteria, *PPD* purified protein derivative, *TB* tuberculosis, *TST* tuberculin skin test

A T-cell-based test may be positive in TB infection as well as TB disease. When positive in a child with a clinical syndrome compatible with TB, a T-cell-based test is supportive of TB as the etiology. However, these tests—regardless of their degree of positivity—cannot distinguish between latent infection and active disease. Determining whether someone has active disease rather than latent infection depends upon findings (e.g. clinical, radiological, laboratory, or endoscopic) consistent with TB disease being present. Furthermore, in children with immunocompromising conditions the sensitivity of T-cell-based tests is decreased. When negative or indeterminate in the setting of a very recent TB exposure or of suspected TB disease (especially one overwhelming the immune system), it may be useful to repeat the T-cell-based test (e.g. in 8–10 weeks) when immune conversion is complete or effective TB treatment reduced the mycobacterial burden. However, a negative T-cell-based (TST/IGRA) test cannot be used to exclude TB infection or disease [43].

### Biochemical markers

Depending on the cutoff levels used, biochemical markers can have a sensitivity and specificity sufficiently high enough to strongly support TB as the cause of pleural or pericardial effusions. Although most studies have been undertaken in adults, their results should also apply in children. In pleural TB, using 40 U/L as the cutoff, the sensitivity of adenosine deaminase (ADA) is approximately 90% and its specificity is around 92% [44, 45]. In pericardial tuberculosis, the sensitivity and specificity of ADA levels 40 U/L are approximately 88 and 83%, respectively [30].

### Microbiological studies

In children with an intrathoracic clinical syndrome consistent with TB, microbiological studies should always be performed as they allow for bacteriological confirmation and for antibiotic susceptibility/resistance testing.

### Specimen collection

**Fig. 3:**
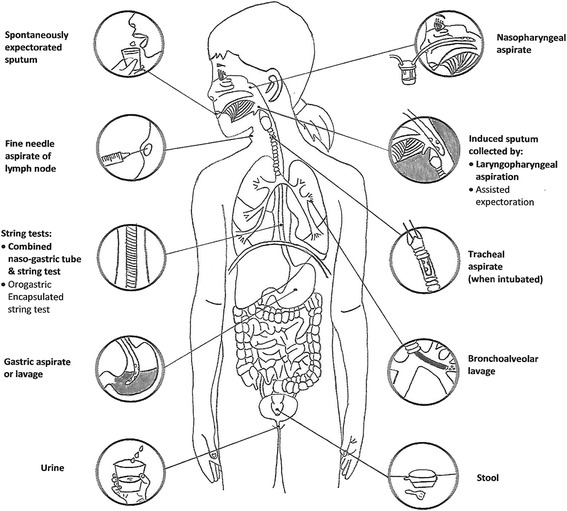
Specimens for bacteriological confirmation of intrathoracic TB in children. Adapted from C.L. Roya-Pabon. Especímenes Respiratorios para el Diagnóstico Microbiológico de las Infecciones Respiratorias. In: Neumología Pediátrica (p. 179), R. Posada-Saldarriaga (Ed.), 2016, Bogotá, Colombia: Distribuna Editorial. Copyright by Distribuna Ltda. [46]. Adapted with permission

*M. tuberculosis* can be detected from various specimens (Fig. 3). Specimen collection should be performed before TB treatment. The specimen collection strategy should include collecting at least two samples (preferably of different specimen types), ensuring high -quality and -quantity of each sample and considering pooling of samples if necessary [47–50]. Early morning respiratory specimens generally have the best yield. Older children (aged ≥10 years) can usually expectorate sputum of adequate quality and volume, *without* coaching or assistance (i.e. spontaneously expectorated sputum). Younger children (aged 5–10 years) can usually expectorate with assistance and the very young (aged <5 years) are unable to expectorate effectively requiring secretions in the laryngopharynx to be suctioned following sputum induction (i.e. induced sputum collected by laryngopharyngeal aspiration) [51]. The term “laryngopharyngeal aspirate” is recommended as this specimen type is collected from the laryngopharyngeal space, it contains less saliva, and is less contaminated by oral microbiota than respiratory secretions passing through the mouth. [21, 46]. Alternatively, lower respiratory secretions that have reached the nasopharynx can be suctioned (known as “nasopharyngeal aspirate”) [51]. Bronchoalveolar lavage should be reserved for children from whom less invasive specimen collection is not attainable, especially as its bacteriological yield is lower than that of serial gastric aspirates [52, 53].

Since young children usually swallow their respiratory secretions, these can be collected by gastric aspiration or lavage (gastric aspiration is preferred). These can also be captured in the esophagus using an intra-esophageal, highly-absorbent nylon yarn, employing as a vehicle for its placement either a gelatin capsule (string test) that is swallowed or a nasogastric tube (combined nasogastric-tube-and-string test). In cooperative children (aged >4 years) able to swallow the gelatin capsule containing the string, the conventional string test is associated with minimal discomfort. In younger children unable to swallow the capsule, the combined nasogastric-tube-and-string test allows two specimens (one gastric aspirate and one string test) to be collected [54, 55]. As young children swallow their sputum, stool may also contain *M. tuberculosis* and a nucleic acid amplification test (NAAT), such as Xpert MTB/RIF (Cepheid, United States of America), on stool can bacteriologically confirm approximately 45% of clinically diagnosed cases of pulmonary TB [56, 57].

In children with enlarged peripheral lymph nodes (usually cervical), a fine needle aspiration biopsy is the specimen of choice, and should be submitted for: (i) mycobacterial testing, i.e. NAAT (Xpert MTB/RIF has a sensitivity of ~83% using culture as reference) and culture; and (ii) pathological studies (cytopathology of aspirate; histopathology of biopsied tissue) [58, 59]. Serosal fluids (e.g. pleural and pericardial) should be collected and submitted for biochemical markers, mycobacterial testing, and cytopathological studies. The diagnostic yield of serosal fluids increases as more types of tests performed. Serosal tissue generally has a higher culture yield and so biopsy (e.g. of the pleura or pericardium) may be justified, especially when drug-resistant TB is suspected (allowing susceptibility testing to be undertaken).

## Mycobacterial detection

### Acid-fast staining and smear microscopy

Acid-fast staining and smear microscopy is a rapid and relatively inexpensive test for detecting acid-fast bacilli (AFB). Unfortunately, the sensitivity of smear microscopy varies greatly based on AFB load. For reliable detection, a sample must contain AFB of at least 1000–10,000 colony-forming units (CFU)/mL [60]. This relatively high detection limit, together with the paucibacillary nature of TB disease in children, contributes to the very low sensitivity of smear microscopy.

Acid-fast stains are also not specific for *M. tuberculosis* complex as they cannot differentiate between mycobacterial species. Nonetheless, in a child with a high pre-test probability of having pulmonary TB, a positive result has a high predictive value, and studies using culture as a reference standard report a very high specificity (~95%) [61–63]. Microscopy’s low sensitivity and inability to differentiate between AFB species (especially relevant for gastric aspirate specimens), means it should not be used as a sole mycobacterial test for detecting *M. tuberculosis*.

### Nucleic acid amplification tests or antigen detection

NAATs are rapid tests that include real-time polymerase chain reaction (RT-PCR) and line probe assays (LPAs) (Additional file 1: Table S2). Recently developed NAATs can also simultaneously detect genes conferring drug resistance, allowing prompt and more appropriate treatment of cases with drug-resistant disease. The fully automated Xpert MTB/RIF test has high sensitivity (pooled estimate 95–96%) in sputum smear-positive samples using culture as a reference standard, but only moderate sensitivity (pooled estimate 55–62%) in smear-negative samples [64]. In 2013, the World Health Organization recommended using Xpert MTB/RIF in samples from children, especially those suspected of multidrug-resistant TB or HIV co-infection [64]. Certain LPAs detect *M. tuberculosis* with/without drug resistance mutations, as well as common NTM, such as *M. avium*, *M. intracellulare*, and *M. kansasii*. GenoType MTBDR*plus*® (Hain Lifescience, Holland) or Genoscholar NTM + MDRTB® (Nipro Europe, Germany) are especially useful for simultaneously detecting isoniazid- and rifampin-resistance in microscopy-positive samples or culture isolates [65–67]. Regarding antigen detection tests the urine lateral flow lipoarabinomannan (LF-LAM) assay may be useful in adolescents with advanced HIV disease and CD4 counts <100 cells/L [68–70]; however, in young children it has poor diagnostic accuracy [71].

### Mycobacterial culture

Mycobacterial cultures have the highest sensitivity and specificity for bacteriological confirmation of intrathoracic TB in children. The limits of detection of liquid and solid media are approximately 10–100 CFU/mL and 50–150 CFU/mL, respectively (versus 100–150 CFU/mL for RT-PCR or 1000–10,000 CFU/mL for fluorescent LED microscopy) [72]. In most prospective studies of children with a clinical diagnosis of probable pulmonary TB, cultures of respiratory specimens are positive in 10–20% of cases. Studies reporting higher rates (i.e. >30%) of culture confirmation are often retrospective and include only children who are hospitalized (probably have more severe disease and better specimen collection strategies) or diagnosed following passive case finding [73]. For definitive species identification following growth in mycobacterial culture, the following methods may be utilized: (i) phenotypic analysis; (ii) antigen tests; (iii) molecular tests such as nucleic acid hybridization probes, matrix-assisted laser desorption/ionization time-of-flight mass spectrometry, and DNA sequencing.

### Histopathology

Histopathological studies should be considered in intrathoracic clinical syndromes compatible with either TB disease or malignancy, especially when bacteriological tests fail to confirm an infectious etiology. Potentially useful tissues to biopsy include lymph nodes, pleura, pericardium and lung. Findings suggestive of TB are numerous granulomas in various developmental stages, some with central caseous necrosis [74]. However, granulomatous inflammation is not sufficiently specific to diagnose TB and differential diagnoses include bacterial (e.g. NTM, nocardiosis), fungal (e.g. histoplasmosis, coccidioidomycosis), helminthic (e.g. schistosomiasis) and, protozoal (e.g. toxoplasmosis) infections, autoimmune diseases (e.g. granulomatosis with polyangiitis), idiopathic etiologies (e.g. sarcoidosis), and foreign bodies.

### Excluding alternative diagnoses

In infants and children, the clinical diagnosis of intrathoracic TB is not always certain, as other disorders can present with similar clinical, radiological, and laboratory abnormalities, or may be present concomitantly. Chronic cough, failure to thrive and prolonged fever for example, have multiple etiologies (Additional file 1: Table S1). It may be possible to exclude some differential diagnoses by using sensitive diagnostic tests or if the child fails a diagnostic-therapeutic trial (i.e., no sustained improvement with appropriate empiric therapy) [75]. Examples of the latter include antibiotics for possible pneumonia, antimalarial agents for fever from presumed malaria, and nutritional support for failure to thrive from suspected under-nutrition. Excluding alternative diagnoses provides further support for a clinical diagnosis of TB disease.

## STEP 3: Screening for risk factors for progression to TB disease

Identifying risk factors for progression from TB infection to disease (Additional file 1: Textbox 2) is important when intrathoracic TB (both pulmonary and extrapulmonary) is suspected. If these are present, this should hasten the diagnostic evaluation; expedite TB treatment (beginning immediately after collecting specimens for microbiological studies) if there are sufficient findings for a presumptive TB diagnosis; and guide preventive therapy in children with TB exposure and infection.

## STEP 4: Follow-up evaluation to further support or exclude TB as the etiology

In very young or immunocompromised children, intrathoracic TB can present acutely; however, in otherwise immunocompetent children, it usually presents as a subacute or chronic illness. In the early stages, there may be insufficient findings to make a presumptive diagnosis, and, even if culture confirmation is attained, this can take weeks. It is therefore critical to perform follow-up evaluations to reassess the patient, whether or not treatment has been initiated, by continuing to reassess steps 1 and 2. On follow-up evaluations, failure to thrive may become more apparent, respiratory symptoms emerge, chest radiography may reveal new or increasing abnormalities, immune-based tests (TST/IGRA) may become positive, and *M. tuberculosis* is detected in respiratory specimens. As most (>90%) children develop disease within the first 12-months of their primary infection, periodic reassessment during the first year of their infection being diagnosed is important.

### Structured diagnostic approaches

The lack of a sensitive diagnostic test for intrathoracic TB means that many diagnostic approaches have been developed. Some are numerical (scoring systems), some hierarchical (case definitions for classification), and others binary (presence or absence of disease). Few have been validated against a gold standard [76]. Although some perform well in advanced disease where clinical and radiological findings are florid, they perform less well in patients with early or mild disease, in young children, and in immunocompromised patients, all of whom are challenging to diagnose [77]. Commonly used approaches have poor agreement with one another and yield highly variable case frequency results from differences in purpose (screening versus diagnosis; patient care versus research versus epidemiological surveillance); healthcare setting (community versus hospital); disease severity (mild versus severe); and prevalence of tuberculosis and/or HIV infection (low versus high) [13].

### Clinical case definitions and management algorithms

**Fig. 4:**
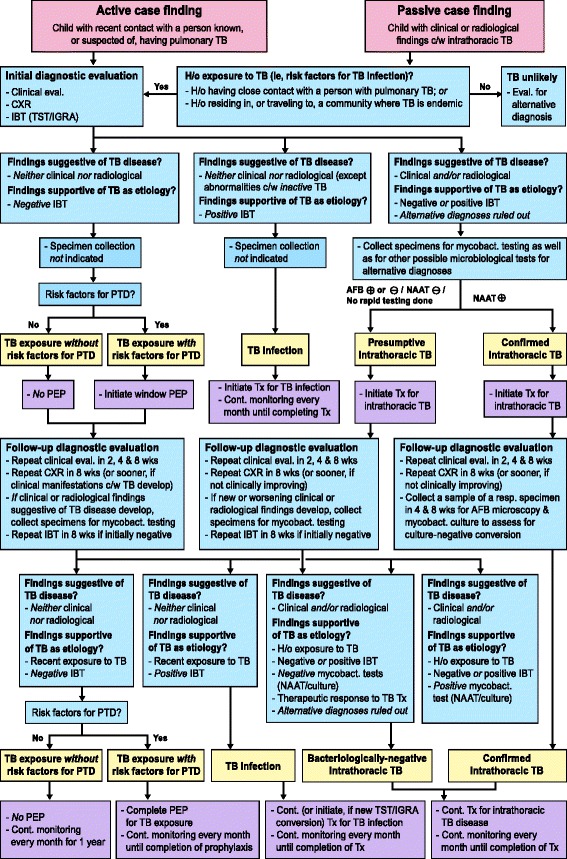
Proposed diagnostic and management algorithm for a child with recent exposure to, or with clinical or radiological findings compatible with TB. AFB: acid-fast bacilli testing; Cont.: continue; c/w: compatible with; CXR: chest radiography; eval.: evaluation H/o: history of; IBT: immune-based test IGRA: interferon-gamma release assay; mycobact.: mycobacterial; NAAT: nucleic acid amplification test; PEP: post-exposure prophylaxis; PTD: progression to TB disease; TB: tuberculosis; TST: tuberculin skin test; Tx: treatment; wks: weeks

Clinical case definitions of TB exposure, infection, and presumptive and confirmed intrathoracic TB in children involve findings suggestive of TB disease (clinical, radiological); findings supportive of TB as the etiology (exposure, immune-based testing, mycobacterial testing, therapeutic response to TB treatment); and risk factors for progression to disease (Table 5). Figure 4 shows an algorithm providing recommendations for diagnosing and managing children with recent exposure to TB (active case finding), or with clinical and/or radiological findings suggestive of TB disease (passive case finding).

**Table 5:** Clinical case definitions and management of TB exposure, infection, and disease in children

Diagnostic classification	Step 1		Step 2			Step 3
	Step 4					
	Findings suggestive of TB disease?		Findings supportive of TB as the likely etiology?			Risk factors? (Management)
	Clinical^a^	CXR^b^	TB exposure (TST/IGRA)	*M. tb* detected	TB treatment response	
TB exposure	None	Normal	Yes (Negative or unavailable)	No	Not applicable	None (no PEP) Yes (consider PEP)
TB infection	None	No signs suggestive of TB disease^c^	Likely (Positive)	No	Not applicable	None (consider LTBI treatment) Yes (provide LTBI treatment)
Presumptive TB	Clinical findings^d^ and/or radiological findings compatible with TB disease		Likely (Positive, may be false negative)	No	Yes	Not applicable (TB treatment)
Confirmed TB			Likely (Positive, may be false negative)	Yes	Yes	Not applicable (TB treatment)

Adapted from C.M. Perez-Velez. Diagnosis of Intrathoracic Tuberculosis in Children. In: Handbook of Child and Adolescent Tuberculosis (p. 168), J.R. Starke and P.R. Donald (Eds.), 2016, New York, NY: Oxford University Press. Copyright by Oxford University Press [15]. Adapted with permission

*Dx* diagnostic, *IGRA* interferon-gamma release assay, *NAAT* nucleic acid amplification test, *PEP* post-exposure prophylaxis, *TB* tuberculosis, *TST* tuberculin skin test, *LTBI* latent TB infection

^a^See “Clinical evaluation” section in text for clinical manifestations suggestive of TB ; ^b^Chest radiograph findings suggestive of TB disease (Fig. 2); ^c^Radiological findings suggestive of inactive TB in a healthy child without symptoms or physical signs compatible with TB include (a) non-enlarged, homogenously calcified regional (parahilar/mediastinal or peripheral) lymph nodes; (b) calcified nodules with round borders in the lung parenchyma; (c) fibrotic scar or discrete linear opacity in the lung parenchyma (±: calcifications within the lesion; or, volume lost, or retraction); and (d) pleural scarring (thickening or calcification). Compare changes with previous imaging studies to ensure that they are radiologically stable; ^d^With TB disease up to 50% of older children with pulmonary TB may have a normal physical exam

### TB exposure [ICD-10: Z20.1]

TB exposure is defined as recent close contact with an adult or adolescent with infectious pulmonary TB (presumptive or bacteriologically confirmed), but without evidence of infection, and lacking clinical or radiological findings suggestive of disease. Not all contacts become infected with TB, but most who do will demonstrate a positive T-cell-based test result within 2–10 weeks [43]. Therefore, in the initial evaluation of a child in a contact investigation, it is not always possible to determine whether a TB exposure has resulted in infection demonstrable by a T-cell-based test. Consequently, until a highly accurate test is developed for detecting an acute TB infection soon after it occurs, it is important to recognize “TB exposure” as a diagnosis, especially in child contacts with risk factors for progression to disease who will benefit from post-exposure prophylaxis. To become infected with *M. tuberculosis*, a susceptible child must inhale droplet nuclei (1–5 microns in diameter) from someone with infectious TB disease who is coughing. This usually involves close (i.e. shared air space in an enclosed environment) contact with an infectious case. Indeed, the longer the duration of exposure and closer the proximity to the case, the higher the risk for transmission. It is thus important to have a high index of suspicion for possible TB infection and to screen for risk factors (Additional file 1: Textbox 2).

### TB infection [ICD-10: R76.11 (by TST); R76.12 (by IGRA)]

TB infection is defined clinically as an infection with any species of the *M. tuberculosis* complex, demonstrated by a positive T-cell-based test (TST and/or IGRA) result, without clinical manifestations or radiological abnormalities consistent with active TB. In a healthy child without symptoms or physical signs compatible with active TB, the following chest imaging findings—when shown to be radiologically stable (i.e., without changes compared to a previous imaging study within the past 4–6 months)—are generally considered indicative of previous TB disease that is currently inactive: (i) non-enlarged, homogeneously calcified regional (parahilar/mediastinal or peripheral) lymph nodes; (ii) calcified nodules with round borders in the lung parenchyma; (iii) fibrotic scar or discrete linear opacity in the lung parenchyma (with or without calcifications within the lesion; or, with or without volume loss, or retraction); and (iv) pleural scarring (thickening or calcification).

### TB disease: clinical syndromes of intrathoracic TB [ICD-10: A15.X]

Intrathoracic TB can affect lung parenchyma, the airways, regional lymph nodes, pleura, and pericardium, while more than one organ may be involved concomitantly. The differential diagnosis of intrathoracic TB is broad, and making a syndromic diagnosis based on clinical, radiological, laboratory, and endoscopic (when indicated) findings helps narrow the list.

### Intrathoracic lymph node disease

Infection and subsequent inflammation of intrathoracic lymph nodes is the pathophysiologic mechanism that determines most of the clinical and radiological findings of intrathoracic TB in children. Many of the radiological patterns in pediatric intrathoracic TB are characterized by intrathoracic lymph node involvement out of proportion to that of the lung parenchyma. Because radiographic density of lymph nodes is similar to that of the heart and consolidated lung, the exact extent of lymph node involvement may be difficult to discriminate on plain radiographs. Their presence is sometimes suggested when the airways are narrowed or displaced. However, chest CT scans can clearly reveal lymph node abnormalities not evident on plain radiography.

Children with isolated, uncomplicated non-calcified, intrathoracic lymphadenopathy are frequently asymptomatic. They are most often found during contact investigations or screening of children with high-risk factors for TB exposure. This radiological pattern may exist in a relatively early state (e.g. subclinical disease) of intrathoracic TB. Chest radiography may reveal one or more enlarged lymph nodes, most often in the right hilum. Subcarinal involvement leads to a splaying of the origins of the two main bronchi. While chest CT scans in these children may find lung parenchymal abnormalities undetectable by plain chest radiography, this investigation is not indicated if they are completely asymptomatic.

Lymph node enlargement, occurring mostly in children aged <5 years, may progress to tracheal or bronchial compression. If complete, this leads to lobar collapse, or if partial to a ball-valve effect causing air-trapping and hyperinflation. Enlarged paratracheal nodes can cause partial tracheal obstruction and stridor. Symptoms vary with the degree of airway compression, from asymptomatic to persistent cough, wheeze or stridor; dyspnea and respiratory distress from extensive atelectasis; or hyperinflation created by pressure from the enlarged lymph nodes on adjacent structures. Chest radiography (especially high-kilovoltage radiography) and CT scans may demonstrate severe narrowing of a bronchus leading to either collapse or hyperinflation, most commonly of the right upper or middle lobes, or the left upper lobe.

### Tracheobronchial disease

Endotracheal and endobronchial disease most often results from bronchogenic spread of TB after a diseased lymph node erodes into the airway, most commonly the left or right main bronchus and bronchus intermedius [24, 28]. Disease may be diffuse or localized with visible granulation tissue [78]. Damaged bronchi may dilate (bronchiectasis) or develop bronchostenosis [79]. Tracheobronchial disease can have an acute, insidious, or delayed onset, with symptoms or physical signs of airway obstruction that depend on the location and severity, including persistent cough, rhonchi, wheeze, stridor, and/or dyspnea. Chest radiography is not sensitive in detecting tracheobronchial disease, unless it is severe or has an associated fibronodular appearance in the lung parenchyma. Bronchiolar disease is revealed on CT scans and may appear as a tree-in-bud pattern or as centrilobular nodules consisting of dilated bronchioles that are thick-walled and filled with mucus. Bronchiectasis is also more easily noted on CT scans, which may show bronchial dilatation and wall-thickening. Bronchoscopy may demonstrate abnormalities suggestive of tracheobronchial disease, including hyperemia, edema, ulcers, masses, stenosis, granulation tissue or caseous lesions [80, 81].

### Parenchymal disease

If inhaled *M. tuberculosis* bacilli are not destroyed immediately by the innate immune response, a small parenchymal focus of infection (primary/Ghon focus) may develop and drain via local lymphatic vessels to regional lymph nodes. Most nodular TB lung disease in children resolves spontaneously and is identified only by radiographic screening during contact investigations. Multiple, focal pulmonary nodules may be seen on chest imaging in the early stages of a TB bronchopneumonia. A child with a solitary pulmonary nodule, with or without associated lymphadenopathy, is most often asymptomatic. Chest radiography may reveal isolated lung opacity with enlarged ipsilateral thoracic lymph nodes, known as a primary/Ghon complex. When lymph node lesions are calcified, it is a Ranke complex. Chest CT scans are more sensitive at detecting small ill-defined airspace nodules that tend to coalesce in some parts, but are different from the discrete, sharply defined micronodules seen in miliary disease.

When the primary infection is poorly contained, mycobacteria replicate and the initial lesion may enlarge (lobar pneumonia). Hilar lymph nodes may also enlarge and sometimes compress or infiltrate contiguous bronchi, most commonly the right or left main bronchus, or bronchus intermedius [24]. If a necrotic hilar lymph node erupts into a bronchus, endobronchial spread leads to patchy or multifocal consolidation of the respective lobe (bronchopneumonia). When enlarged hilar lymph nodes are also compressing the bronchus, the endobronchial spread may cause distal expansion and dense consolidation of the entire lobe (expansile pneumonia) displacing the trachea, bowing the fissures and forming focal cavities. Cavities are uncommon in children, occurring predominantly in infants with extensive, uncontained disease or in adolescents with “adult-type” disease. Chest radiography and CT scans may reveal an oval-shaped lucency that is either isolated or within a consolidation or nodule, with walls that may be either thin or thick. In older children and adolescents there may be multiple cavities, located typically in the apical segments of the upper or lower lobes [9].

### Pleural disease

TB pleural effusions typically occur 3–6 months after a primary infection and are usually unilateral, mostly resulting from a delayed-type hypersensitivity reaction to *M. tuberculosis* antigens that leaked into the pleural space from a subpleural primary focus. Pleural thickening is a common component of the primary complex, but it rarely leads to a significant effusion. Large effusions are seen more often in older children (age >5 years) and adolescents. The child most often presents with pleuritic chest pain (58%), cough (80%) and fever (67%) [82]. Chest radiography will reveal a homogeneously opacified fluid level, with pulmonary parenchymal abnormalities (usually consolidation) and intrathoracic lymphadenopathy often becoming visible post-drainage [21]. Chest ultrasonography is useful in determining the nature and quantity of the effusion and detecting early loculations and septations. Chest CT scans are useful in cases with complicated pleural effusion, detecting associated parenchymal lesions and intrathoracic lymphadenopathy, and differentiating between pleural thickening and a chronic loculated effusion or empyema. TB pleural fluids are most often exudative with lymphocytic pleocytosis. Because of its protein-rich nature, care must be taken to not remove too much pleural fluid in a severely malnourished child because this can acutely worsen the child's oncotic pressure. TB empyema has also been described [83], where pleural fluid is purulent [84]. Chylothorax is a rare type of pleural effusion resulting from disruption or obstruction of the thoracic duct (or its tributaries), leading to lymphatic fluid (chyle) leakage into the pleural space. The pleural fluid typically has a milky white appearance, and is predominantly lymphocytic with elevated levels of triglycerides (>1.2 mmol/L) [85].

### Pericardial disease

TB is one of the most common causes of pericardial effusion in children in TB-endemic countries, and approximately 1–4% of children with TB develop pericarditis [86]. It has three main presentations: pericardial effusion (the most common), constrictive pericarditis, and a combination known as effusive-constrictive disease. It most frequently occurs after an infected contiguous subcarinal lymph node infiltrates the pericardium. It can also arise from lymphohematogenous dissemination of *M. tuberculosis*. HIV infection predisposes a patient to such disseminated disease, and is associated with greater severity of pericardial TB [87]. Children with TB pericarditis usually present with symptoms and signs of heart failure, including persistent cough (70%), dyspnea (77%), chest pain (30%), hepatomegaly (77%), elevated jugular venous pressure (7%), soft heart sounds, and a pericardial friction rub (18%), in addition to fever (52%), night sweats, failure to thrive (36%), fatigue, and malaise [88]. Chest radiography typically reveals cardiomegaly with a globular heart silhouette (91%). Echocardiography is the most sensitive study to confirm a pericardial effusion, and may reveal associated mediastinal lymphadenopathy or other complications.

### Disseminated/miliary disease

Miliary lung disease results from a TB lesion infiltrating into a blood vessel, leading to hematogenous dissemination [89]. The temporal pattern of miliary disease is usually acute, but it can also present with a delayed onset. Pulmonary involvement and respiratory symptoms occur relatively late in the disease. Given the multisystem involvement, presenting symptoms may include cough (72%), dyspnea, diarrhea and vomiting (33%), irritability, headache, convulsions, hepatomegaly (82%), splenomegaly (54%), lymphadenopathy (46%), fever (39%), chills, loss of appetite and failure to thrive (40%), fatigue, generalized weakness, decreased activity, and malaise. The main complication is TB meningitis [89]. Chest radiography may reveal innumerable rounded micronodules (≤3 mm in diameter) scattered diffusely throughout both lungs, but in the initial stages of disseminated disease the radiological abnormalities may not be apparent (9%) [79, 89]. Often these nodules are best seen on the lateral projection of the chest radiograph in the retrocardiac area.

## Conclusions

Using currently available tools, a systematic diagnostic approach to the child with recent exposure to, or with clinical or radiological findings compatible with, TB can allow the clinician to classify most patients into one of the major diagnostic categories of TB exposure, infection, or disease. In cases of TB exposure and infection, identifying risk factors for progression to disease helps hasten diagnostic evaluation and initiating appropriate prophylaxis or treatment when indicated.

## Additional file

Additional file 1:
**Table S1.** Differential diagnosis of chronic cough in children. **Table S2.** Nucleic acid amplification tests for detecting *Mycobacterium tuberculosis* complex and genes encoding targets of mutations conferring drug resistance. **Table S3.** Differential diagnosis of clinical-radiological syndromes associated with intrathoracic TB in children. **Textbox 1.** Spectrum of possible organ involvement in TB disease. **Textbox 2.** Risk factors for TB infection in children. (PDF 617 kb)

## Abbreviations

ADA: Adenosine deaminase
AFB: Acid-fast bacilli
BCG: Bacille Calmette-Guerin
CFU: Colony-forming unit
CT: Computed tomography
DNA: Deoxyribonucleic acid
HIV: Human immunodeficiency virus
ICD-10: International statistical classification of diseases and Related health problems, 10th revision
IGRA: Interferon-gamma release assay
LDH: Lactate dehydrogenase
LED: Light-emitting diode
LF-LAM: Lateral flow lipoarabinomannan
LPA: Line probe assay
*M.*: *Mycobacterium*
MDR: Multi-drug-resistant
NAAT: Nucleic acid amplification test
NTM: Nontuberculous mycobacteria
PPD: Purified protein derivative
RT-PCR: Real-time polymerase chain reaction
TB: Tuberculosis
TST: Tuberculin skin test
