# Supplementary material for: Tuberculosis exposure, infection and disease in children: a systematic diagnostic approach
Source: Pneumonia (Nathan). 2016 Nov 24;8:23. doi: 10.1186/s41479-016-0023-9 (PMC5471717; doi:10.1186/s41479-016-0023-9)
Supplement: Additional file 1: — Table S1. Differential diagnosis of chronic cough in children. Table S2. Nucleic acid amplification tests for detecting Mycobacterium tuberculosis complex and genes encoding targets of mutations conferring drug resistance. Table S3. Differential diagnosis of clinical-radiological syndromes associated with intrathoracic TB in children. Textbox 1. Spectrum of possible organ involvement in TB disease. Textbox 2. Risk factors for TB infection in children. (PDF 617 kb) [file 41479_2016_23_MOESM1_ESM.pdf]

SUPPLEMENTARY ON-LINE MATERIAL

Supplementary Table 1. Differential diagnosis of chronic cough in children

Cause	Nature of Cough	Associated Features	Response to Therapy
**Infectious diseases**			
**Pulmonary tuberculosis**	Wet, persistent & unremitting cough. Mucopurulent sputum; rarely bloody (with underlying bronchiectasis or cavitary disease).	Variable fever. Failure to thrive / malnutrition. Exposure to TB; fatigue or decreased activity/playfulness; mediastinal, hilar lymphadenopathies.	Not resolved with trial of antibiotics. Significant improvement with TB treatment
**Lymph node tuberculosis**	Dry, persistent & unremitting cough.	Variable fever. Failure to thrive / malnutrition. Subacute onset; wheezing or stridor.	No response to bronchodilators or antibiotics; Significant improvement with TB treatment
**Recurrent viral respiratory tract infection**	Acute-onset cough, initially dry then wet	Recurrent fever. Normal nutritional status. Especially infants; coryza, sore throat; improvement between episodes.	Delayed recovery, with back-to-back relapses; no response to antibacterials.
**Bronchiolitis**	Cough marked at onset, with steady improvement.	Fever at onset, then resolves. Normal nutritional status. Wheezing; infants.	Responsive to general supportive measures.
**Pertussis-like syndrome** (e.g., *Bordetella*; *Chlamydophila*; *Mycoplasma*; resp. viruses)	Intractable, loud, dry, paroxysmal cough; not always with inspiratory whoop. Small amounts of viscid clear sputum.	Fever at onset, then resolves. Normal nutritional status. Not immunized; subconjunctival hemorrhages.	Can be very slow to resolve, antibiotics have limited impact
**Protracted bacterial bronchitis** (e.g., *S. pneumoniae*; *H. influenzae*; *M. catarrhalis*)	Persistent wet-moist cough with delayed recovery. Mucopurulent sputum.	Fever at onset, then resolves. Normal nutritional status. Especially young children (< 5 y.o.), who otherwise appear well.	Resolves with prolonged (>2-weeks) oral antibiotics
**Recurrent bacterial pneumonia**	Acute-onset cough, then improvement, then relapse. Mucopurulent sputum.	Recurrent fever. Failure to thrive / malnutrition. Improvement between episodes; may be HIV-related.	Resolves with antibiotics
**Chronic bacterial rhinosinusitis**	Persistent cough, worse when lying down.	Fever at onset, then resolves. Normal nutritional status. Postnasal drainage.	Resolves with appropriate treatment
**Allergic disease**			
**Allergic chronic**	Variable cough, worse	Fever absent unless assoc. with	Can be controlled with

**rhinosinusitis**	when lying down.	secondary infection. Normal nutritional status. Nasal congestion & postnasal drainage; frequent clearing of throat.	ongoing combination of allergen avoidance, medications, immunotherapy.
**Cough-dominant asthma**	Recurrent episodes of cough, usually dry, worse at night. May be productive of thick/mucoid sputum.	Fever absent unless assoc. with secondary infection. Normal nutritional status. May be accompanied by wheezing & dyspnea.	Responsive to bronchodilators & glucocorticoids
**Mucociliary disorders**			
**Cystic fibrosis**	Persistent wet cough. Copious viscid mucopurulent sputum.	Variable fever. Failure to thrive / malnutrition. Begins in early childhood; bronchiectasis; frequent wheezing; clubbing; generally Caucasian.	Acute exacerbations resolve with antibiotics; chronic cough ameliorated with daily pulmonary hygiene therapies.
**Primary ciliary dyskinesia**	Persistent moist cough. Mucoid or purulent sputum.	Fever absent (even sometimes during exacerbations). Normal nutritional status. Bronchiectasis; occasional wheezing; chronic rhinosinusitis, recurrent otitis media; may have situs inversus.	Acute exacerbations resolve with antibiotics; chronic cough ameliorated with daily pulmonary hygiene therapies.
**Aspiration syndromes**			
**Gastroesophageal reflux disease** (GERD)	Dry cough with variable persistence, worse at night, sometimes associated with stridor and wheezing; hoarseness.	Fever absent unless assoc. with aspiration-related lower respiratory tract infection. Failure to thrive (especially in severe cases). Children with neurologic abnormalities are at greater risk for aspiration complications (pneumonitis/pneumonia).	Usually responsive to dietary and medical measures.<br>Dysphagia cases may be responsive to swallow therapy;
**Retained foreign body**	Persistent cough.	Fever absent unless assoc. with secondary infection. Normal nutritional status. Especially toddlers; choking episode at onset of aspiration.	Removal of aspirated foreign body by rigid bronchoscopy.
**Others**			
**Lymphoid interstitial pneumonitis (LIP)**	Persistent cough.	Variable fever. Variable nutritional status. HIV-infected; parotid enlargement; persistent generalized lymphadenopathy; clubbing.	Responsive to corticosteroids.

**Tracheomalacia (TM)**	Brassy cough, expiratory stridor, wheezing. Thin, clear, scarce sputum.	Fever absent unless assoc. with secondary infection. Normal nutritional status. Laryngeal clefts, tracheoesophageal fistula, bronchomalacia.	Mild congenital TM improves as the infant grows. Severe TM requires surgical care.
**Congestive heart failure**	Persistent cough; worse at night. Thin, frothy sputum.	Fever absent unless assoc. with secondary infection. Failure to thrive / malnutrition. Pulm. edema; exercise intolerance & easy fatigue; respiratory distress with tachypnea; hepatomegaly.	Depends on the underlying etiology.

LIP: lymphoid interstitial pneumonitis; **TB**: tuberculosis; **TM**: tracheomalacia

Adapted from C.M. Perez-Velez. Diagnosis of Intrathoracic Tuberculosis in Children. In: Handbook of Child and Adolescent Tuberculosis (p. 162-165), J.R. Starke and P.R. Donald (Eds.), 2016, New York, NY: Oxford University Press. Copyright by Oxford University Press [15]. Adapted with permission.

Supplementary Table 2. Nucleic acid amplification tests for detecting *Mycobacterium tuberculosis* complex and genes encoding targets of mutations conferring drug resistance

**NAAT**	**Type**	**Gene Encoding Target**	**Drug-Resistance: Gene Encoding Target of Mutations**	**Analytical Sensitivity (Limit of Detection)** (CFU/mL)	**Recommended Smear Status for Detecting *M. tb***	**Turnaround Time**	**Required Risk Level of Laboratory**	**Year of Release**	**Endorsement/ Approval**
**GenoType MTBDR*plus*** version 2 (Hain Lifescience)	Line probe assay	- *M. tb* complex: 23SrRNA	- Rmp: *rpoB*<br>- INH: *katG*; *inhA*	1000-10,000	Only positive	4-6 hours (manual)	High	2012 (version 1 in 2004)	WHO
**GenoType MTBDR*sl***	Line probe	- *M. tb* complex: 23SrRNA	- Fluoroquinolones:	1000-10,000	Only positive	4-6 hours (manual)	High	2012 (version 1	WHO

version 2 (Hain Lifescience)	assay		*gyrA* - Aminoglycosides & Cyclic Peptides: *rrs* - Emb: *embB*					in 2009)	
**Genoscholar NTM + MDRTB** (Nipro)	Line probe assay	- *M. tb* complex - *M. avium* - *M. intracellulare* - *M. kansasii*	- Rmp: *rpoB* - INH: *katG*; *inhA*	1000-10,000	Only positive	4-6 hours (manual)	High	2012	WHO
**COBAS TaqMan MTD Test (Roche)**	RT-PCR	- *M. tb* complex: 16S rRNA	N/A	20	Only positive	6.5 hours (automated)	Moderate	2010	FDA
**Xpert MTB/RIF** (Cepheid)	RT-PCR	- *M. tb* complex: *rpoB*	- Rmp: *rpoB*	100-150	Positive & Negative	1-2 hours (automated)	Low	2010	WHO FDA
**Xpert MTB/RIF Ultra** (Cepheid)	RT-PCR	- *M. tb* complex: *rpoB*	- Rmp: *rpoB*	10-100	Positive & Negative	1-2 hours (automated)	Low	? 2017	N/A

CFU: colony-forming units; **Emb**: ethambutol; **FDA**: Federal Drug Agency; **INH**: isoniazid; ***M. tuberculosis****:* *Mycobacterium tuberculosis*; **NAAT**: nucleic acid amplification test; **RT-PCR**: real-time polymerase chain reaction; **TAM**: transcription-mediated amplification; **WHO**: World Health Organization.

Supplementary Table 3: Differential diagnosis of clinical-radiological syndromes associated with intrathoracic TB in children

**Clinical-Radiological Syndrome**	**Differential Diagnosis**	
	**Infectious**	**Non-Infectious**

**Lymph Node Disease**		
Lymphadenopathies,<br>Mediastinal/Hilar/Paratracheal<br>- Noncalcified<br>- Calcified (Ranke)	Mycobacteria: *M. tb*; MAC<br>Fungi: *Histoplasma*; *Coccidioides*<br>Bacteria: *B. pertussis*, *B. henselae* (cat scratch disease)<br>Viruses: HIV; measles<br>Parasite: *Toxoplasma*	Tumors: lymphoma (Hodgkin's & non-Hodgkin's); lymphangioma; lymphosarcoma; leukemia<br>Others: sarcoidosis; hyperplastic thymus (or even normal thymus in infant); teratoma
**Parenchymal Lung Disease**		
Solitary pulmonary nodule<br>- Noncalcified<br>- Calcified	Mycobacteria: *M. tb* (primary Ghon focus); MAC<br>Fungi: *Histoplasma*; *Aspergillus*; *Coccidioides*	Malignant tumors: sarcoma; lymphoma germ cell tumor<br>Others: hamartoma; arteriovenous malformations
Multiple focal pulmonary nodules<br>- Noncalcified<br>- Calcified	Mycobacteria: *M. tb*; MAC<br>Bacteria: septic pulmonary emboli due to *S. aureus*; *Nocardia*<br>Atypical Bacteria: *M. pneumoniae*; *Legionella; C. psittaci*<br>Fungi: *Aspergillus*; *Cryptococcus*; *Histoplasma*; Coccidioides; *Candida*<br>Viruses: CMV; VZV; HSV; HPV	Malignant tumors: sarcoma; lymphoma; Wilms' tumor<br>Autoimmune: granulomatosis with polyangiitis (Wegener's)<br>Others: hamartoma; recurrent pulmonary aspirations; hypersensitivity pneumonitis; pulmonary hemosiderosis
Diffuse pulmonary micronodules	Bacteria: streptococci & *Listeria* (esp. neonates)<br>Mycobacteria: *M. tb* (miliary disease)<br>Fungi: *Aspergillus*; *Cryptococcus*; *Histoplasma*; *Coccidioides*; mucormycosis	Malignant tumors: sarcoma<br>Benign tumors: inflammatory myofibroblastic tumor<br>Others: toxic fume inhalation; alveolar hemorrhage syndrome
Pneumonia		
Acute lobar pneumonia	Viruses: RSV; influenza; parainfluenza; adenovirus; metapneumovirus<br>Bacteria: *S. pneumoniae*; *S. aureus*; *S. pyogenes*; *H. influenzae*; *K. pneumoniae*	Congenital malformations: bronchopulmonary sequestration; bronchogenic cyst; tracheoesophageal fistula<br>Others: pulmonary infarct

	Mycobacteria: *M. tb*	
Subacute lobar pneumonia	Atypical Bacteria: *M. pneumoniae*, *C. pneumoniae*<br>Viruses: adenovirus<br>Mycobacteria: *M. tb*; MAC; *M. abscessus*	Aspiration syndromes: aspiration pneumonia
Chronic lobar pneumonia	Bacteria: *Actinomyces*; *B. anthracis*; anaerobes<br>Atypical Bacteria: *M. pneumoniae*; *C. pneumoniae*; *F. tularensis*<br>Mycobacteria: *M. tb*; MAC; *M. abscessus*<br>Fungi: *Histoplasma*; *Coccidioides*; *Blastomyces*; *Cryptococcus*<br>Parasites: *Paragonimus*	Aspiration syndromes: aspiration pneumonia; foreign body
Cavitary pulmonary disease<br>- Solitary cavity<br>- Multiple cavities	Bacteria: *S. aureus*; *K. pneumoniae*; *Actinomyces*; *Nocardia*; anaerobes<br>Mycobacteria: *M. tb* (progressive Ghon focus)<br>Fungi: *Histoplasma*; *Coccidioides*; *Aspergillus*; *Pneumocystis*<br>Viruses: HPV; influenza; measles<br>Parasites: *Paragonimus*; *E. histolytica*; *Echinococcus*	Malignant tumors: lymphoma<br>Benign tumors: inflammatory pseudotumor<br>Autoimmune: granulomatosis with polyangiitis (Wegener's)<br>Others: cystic bronchiectasis; congenital; sarcoidosis; traumatic pneumatocele
**Airway Disease**		
Subacute/Chronic bronchitis	Virus: post-viral reactive airways disease<br>Atypical Bacteria: *M. pneumoniae*; *C. pneumoniae*<br>Bacteria: protracted bacterial bronchitis (*H. influenzae; S. pneumoniae*; *M. catarrhalis)*<br>Mycobacteria: *M. tb*<br>Fungi: *Aspergillus* (e.g., ABPA)	Obstructive airway diseases: asthma; cystic fibrosis<br>Others: bronchomalacia; gastroesophageal reflux; airway irritation by smoke or air pollution
Endobronchial granulomas	Mycobacteria: *M. tb*; MAC	Autoimmune: eosinophilic granulomatosis

	Viruses: HPV	with polyangiitis<br>Others: sarcoidosis
Tree-in-bud pattern	Mycobacteria: *M. tb*; MAC<br>Fungi: *Aspergillus*; *Pneumocystis*<br>Viruses: RSV; parainfluenza	Mucociliary disorders: cystic fibrosis; primary ciliary dyskinesia<br>Autoimmune: rheumatoid arthritis
Bronchiectasis	Sequelae of chronic/severe airway infections due to the following:<br>Bacteria: *S. aureus*; *K. pneumoniae*; *B. pertussis*<br>Mycobacteria: *M. tb*; MAC<br>Viruses*:* influenza; RSV; adenovirus; measles<br>Fungi: *Aspergillus*	Mucociliary disorders: cystic fibrosis; primary ciliary dyskinesia<br>Aspiration syndromes: recurrent pulmonary aspiration; foreign body
**Pleural Disease**		
Pleural effusion	Bacteria: *S. pneumoniae*; *S. aureus*; *H. influenzae*; *P. aeruginosa*; *Actinomyces*<br>Atypical Bacteria: *M. pneumoniae*<br>Virus: adenovirus; influenza<br>Mycobacteria: *M. tb*<br>Fungi: *Aspergillus*; *Histoplasma*<br>Parasite: *Paragonimus*	Malignant tumors: lymphoma<br>Autoimmune: systemic lupus erythematosus<br>Vascular: lymphatic disorders
Chylothorax	Mycobacteria: *M. tb* (mediastinal lymph node disease)<br>Fungi: *Histoplasma* (mediastinal lymph node disease)	Malignant tumors: lymphoma; teratoma; sarcoma; neuroblastoma<br>Others: thoracic duct injury; congenital malformation; sarcoidosis

ABPA: allergic bronchopulmonary aspergillosis; ***B. anthracis****: Bacillus anthracis;* ***B. henselae:*** *Bartonella henselae;* ***B. pertussis****: Bordetella pertussis;* **CMV**: cytomegalovirus; ***C. pneumoniae***: *Chlamydophila pneumoniae*; ***C. psittaci***: *Chlamydia psittaci*; ***E. histolytica***: *Entamoeba histolytica*; ***F. tularensis***: *Francisella tularensis*; ***H. influenzae***: *Haemophilus influenzae;* **HIV**: human immunodeficiency virus; **HPV**: human papillomavirus; **HSV**: herpes simplex virus; ***K. pneumoniae***: *Klebsiella pneumoniae*; ***M. abscessus***: *Mycobacterium abscessus*; ***M. catarrhalis***: *Moraxella catarrhalis*; ***M. pneumoniae***: *Mycoplasma pneumoniae*; ***M. tb:*** *Mycobacterium tuberculosis*; **MAC**: Mycobacterium avium complex; ***P. aeruginosa***: *Pseudomonas aeruginosa*; **RSV**: respiratory syncytial virus; ***S. aureus***: *Staphylococcus aureus*; ***S. pneumonia***: *Streptococcus pneumonia*; ***S. pyogenes***: *Streptococcus pyogenes*; **VZV**: varicella zoster virus

Adapted from C.M. Perez-Velez. Diagnosis of Intrathoracic Tuberculosis in Children. In: Handbook of Child and Adolescent Tuberculosis (p. 157-159), J.R. Starke and P.R. Donald (Eds.), 2016, New York, NY: Oxford University Press. Copyright by Oxford University Press [15]. Adapted with permission.

Supplementary Textbox 1. Spectrum of possible organ involvement in TB disease

- **Respiratory System** (including chest): lungs; pleura; larynx; nasopharynx; sinuses.
- **Reticuloendothelial System**: lymph nodes; bone marrow; liver; spleen.
- **Cardiovascular System**: pericardium; myocardium.
- **Central Nervous System** (including head, neck, ears, and eyes): meninges; brain; orbit; optic neuritis; retina; uvea; sclera; conjunctiva.
- **Musculoskeletal System**: bone; joint; bursa; muscle.
- **Urinary System**: kidneys; ureters; bladder.
- **Alimentary/Digestive System** (including abdomen): oral cavity; esophagus; intestine; peritoneum; liver; gallbladder.
- **Endocrine System**: pancreatic; adrenal; thyroid; parathyroid; breast; pituitary; testicular; ovarian.
- **Reproductive/Genital System**: uterine; oviduct; vulva; prostate; epididymis; penis.
- **Integumentary System**: skin.

Adapted from C.M. Perez-Velez. Diagnosis of Intrathoracic Tuberculosis in Children. In: Handbook of Child and Adolescent Tuberculosis (p. 151), J.R. Starke and P.R. Donald (Eds.), 2016, New York, NY: Oxford University Press. Copyright by Oxford University Press [15]. Adapted with permission.

Supplementary Textbox 2. Risk factors for TB infection in children

Close contact with an adult or adolescent with confirmed or suspected pulmonary TB

Close contact with an adult or adolescent known to have TB infection

Having one of the following risk factors for TB infection, or having a close contact with an adult or adolescent having one of them:

- Birth in, or travel to, an area of the world that is endemic for TB (especially during the last five years)
- Residing or working with persons who are at high risk for TB in a congregate setting such as a:
    - healthcare institution that cares for high-risk patients (if infection control is not adequate), including:
        - general and psychiatric hospitals and clinics
        - urgent care centre
        - physician's office
        - skilled nursing facility
    - long-term residential facilities

    - correctional institution
    - homeless shelter
- Homelessness
- Illicit drug use, particularly by injection
